# Assessing transcriptomic responses of *Salmonella* Infantis in the presence of poultry litter

**DOI:** 10.1128/spectrum.03307-25

**Published:** 2026-03-10

**Authors:** Ruby Paudel, Dianna Bourassa, Sabin Poudel

**Affiliations:** 1 Department of Poultry Science, Auburn University 708995, Auburn, Alabama, USA; Earlham Institute, Norwich, United Kingdom

**Keywords:** *Salmonella *Infantis, litter, survival, transcriptomics

## Abstract

Litter is a major source for harboring and transmitting *Salmonella* from one flock to another flock. The objective of this experiment was to elucidate the persistence mechanism of *S*. Infantis by analyzing transcriptomic changes in the presence of litter. To achieve this, four treatments were arranged: (1) BHI, (2) BHI+Litter, (3) PBS, and (4) PBS+Litter. Each treatment was inoculated with *S*. Infantis, and RNA was extracted. Quality, quantity, and integrity of RNA were assessed and then subjected to Illumina sequencing. HISAT2 was used to align reads to the reference genome, HTSeq to obtain read counts, and DESeq to study differential gene expression. The compared treatments were BHI vs BHI+Litter, PBS vs PBS+Litter, and PBS vs BHI. Starvation stress was pronounced in bacteria with upregulated expression of *rpoS* and *dksA* genes in nutrient-poor media, like PBS, in comparison to BHI and litter treatments. Additionally, the genes *fad, eutS,* and *pduF,* associated with alternative carbon utilization, were upregulated in PBS compared to PBS+Litter and BHI. Compared to non-litter media, alternative respiration system genes *dmsA, dmsB, dmsC*, *ttrB, ttrC*, *fdnH,* and *fdnG* upregulation in litter highlights *S*. Infantis’ ability for metabolic adaptation to oxygen-limited environments. Furthermore, virulent genes such as *invA* and *sopD* were found to be upregulated in BHI in comparison to BHI+Litter. In contrast, genes associated with amino acid-dependent acid resistance mechanisms including *adiA, cadA,* and *cadB* were downregulated in BHI in comparison to BHI+Litter. In conclusion, *S*. Infantis upregulated stress response and metabolic genes to survive in challenging environments.

**IMPORTANCE:**

Poultry litter plays a pivotal role in the persistence, proliferation, and transmission of *Salmonella*. Recent studies have reported the increased prevalence of *Salmonella* Infantis in the broiler houses and chicken meat in the United States and Europe. Moreover, *Salmonella* strains could persist in the recycled litter despite undergoing several treatment methods. In commercial production systems, it is a common practice to reuse poultry litter for raising multiple broiler flocks in the United States. Therefore, it is crucial to understand the mechanism of the survival of bacteria in reused litter to curb the prevalence of *Salmonella*. The objective of this study was to determine the transcriptional variations in *S*. Infantis in the presence and absence of litter. The findings from this study will provide insights into the potential persistence mechanism of *S*. Infantis within the litter environments.

## INTRODUCTION

Salmonellosis is one of the leading food-borne illnesses in the United States, which is caused by the gram-negative bacterium *Salmonella* (1). *Salmonella* comprises more than 2600 serovars that account for typhoidal and non-typhoidal illnesses in humans (2, 3). Non-typhoidal Salmonellosis (NTS) is a zoonotic disease caused by *S. enterica* species that is transmitted from animals to humans (4). In the United States, NTS is estimated to result in 1.35 million illnesses yearly, with 19,336 hospitalizations and 378 deaths (5, 6). More than 1,500 serovars contribute to the outcome of NTS in humans (7). These bacteria are commonly found in the gastrointestinal tract of food-producing animals and wild birds, which serve as the reservoirs of infection (8). Although NTS is often limited to diarrheal illness, sometimes, it progresses to complications such as bacteremia and meningitis (9). In humans, infections can be acquired due to the consumption of contaminated meat, eggs, dairy, and agricultural produce (8, 9). Thus, studying the NTS in food-producing animals is crucial to understanding its transmission mechanism.

Among different food-producing animals, poultry serves as the primary reservoir for transmitting NTS serotypes to humans (10). The recent source attribution analysis of NTS in humans from 2003 to 2018 predicted that approximately one-third of the illnesses originated from chicken (11). Additionally, the data from the National Outbreak reporting system indicate that along with other *Salmonella* serotypes (Enteritidis and Typhimurium) that cause human hospitalization, *S*. Infantis is also consistently causing human hospitalization (12). In alignment with this, a surveillance report from the Food Safety and Inspection Service (FSIS) also showed an increasing trend in the isolation of *S*. Infantis in chickens from 2016 to 2021 in the United States (13). Taken together, these data indicate that *S*. Infantis could become one of the primary causes of human Salmonellosis in the near future if the prevalence of *S*. Infantis is not checked. Several environmental factors contribute to the growth and spread of *Salmonella*, and poultry litter is one of the key factors associated with the contamination and persistence of *Salmonella* in the poultry production system (14). Furthermore, recent studies have recognized *S*. Infantis as the predominant serovar in broiler chickens, highlighting the need to understand its ecology and persistence/survival mechanism in poultry environments (15, 16).

Poultry litter is composed of bedding material with feces, feathers, and feed, which provides a favorable environment to host diverse microorganisms (17, 18). A recent study in broiler houses across the United States reported the persistence of several serotypes of *Salmonella* in the litter (19). Additionally, *Salmonella* strains could persist in the recycled litter despite undergoing several treatment methods (20). In a commercial production system, it is a common practice to reuse poultry litter for raising multiple broiler flocks in the United States (21, 22). So, the litter could possibly act as a transmitter of *Salmonella* from past flocks to subsequent flocks. Therefore, it is crucial to understand the mechanism of the survival of bacteria in reused litter. This study was conducted to investigate the persistence mechanism of *S*. Infantis in litter, with the focus of understanding the roles of various genes in bacterial survival by using RNA sequencing under different conditions.

## MATERIALS AND METHODS

### Bacterial strain and inoculum preparation

*Salmonella* Infantis BAA 1675 was cultured on Xylose Lysine Deoxycholate (XLD) agar and incubated at 37°C for 24 h, and colonies were then sub-cultured into Brain Heart Infusion (BHI) media (BD Diagnostics, Catalog 299070). A spectrophotometer (VWR V-1200 Visible Spectrophotometer, Catalog 10037-434) was used to measure the optical density (OD_600_ = 0.558), and bacteria were enumerated (2.5 × 10^9^ CFU/ mL) after serial dilution. Thus, the prepared bacterial inoculum was further used for the bacterial inoculation.

### Preparation of treatments and bacterial inoculation

Litter samples were collected from pen houses located at the Charles C. Miller Jr. Poultry Research Center of Auburn University. To confirm the absence of *Salmonella* in litter samples, litter samples were enriched in Buffered Peptone Water (BPW) media for 24 h at 37℃ which was followed by culture on XLD plates (14, 22). Additionally, the genomic DNA from the litter samples (washed with InhibitEX [Qiagen, Catalog 19593]) was extracted using QIAamp Power Fecal Pro DNA Kit (Qiagen, Catalog 51804), and the *Salmonella* specific gene *invA* was used for PCR, verifying the absence of *Salmonella* (23, 24). The confirmed *Salmonella* negative litter was used for the preparation of the treatments. Treatments were arranged in 2 × 2 factorial arrangements with each treatment having four replications. The treatments were brain heart infusion (BHI) broth, BHI with litter (BHI+Litter), phosphate-buffered saline (PBS), and PBS with litter (PBS+Litter). The prepared bacterial inoculum was added to the sterile whirl-pack plastic bags with or without litter to make the final concentration of *S*. Infantis 2.5 × 10^7^ Cfu/mL; thus, prepared samples were incubated at 37°C for 24 h (Table 1). Following incubation, the culture solution was subjected to centrifugation at an RCF of 12,506 × *g* for 15 min to make cell pellet. Thus, the obtained cell pellet was resuspended in 1,000 μL of RNA Later (Invitrogen, Thermo Fisher Scientific, Catalog: AM7021) and stored at −80°C until further processing.

**TABLE 1:** Bacterial inoculation and treatment preparation: experimental layout for *S*. Infantis transcriptome analysis^*a*^

SN	Treatment	Treatment composition	Inoculum added	Final *S*. Infantis conc
1	BHI+Litter	20 gm litter + 100 mL BHI	1,000 μL	2.5 × 10^7^ CFU/ mL
2	PBS+Litter	20 gm litter + 100 mL PBS	1,000 μL	2.5 × 10^7^ CFU/ mL
3	BHI	20 mL BHI	200 μL	2.5 × 10^7^ CFU/ mL
4	PBS	20 mL PBS	200 μL	2.5 × 10^7^ CFU/ mL

^*a*^ A total of 1,000 μL of 2.5 × 10^9^ CFU/ mL of the prepared inoculum was added in medium with litter and 200 μL of 2.5 × 10^9^ CFU/mL of the prepared inoculum was added in medium without litter such that final concentration of bacteria was 2.5 × 10^7^ CFU/mL across all treatments.

### RNA extraction and library preparation

Cell pellets were resuspended in RNA lysis buffer, transferred into ZR Bashing Bead Lysis Tube (0.1 & 0.5 mm), and samples were homogenized (VWR 4-Place Mini Bead Mill, Catalog 432-0366) for 2 min. Following the bead beating, the RNA was extracted using the RNeasy Mini Kit (Qiagen, Catalog 74106) following the manufacturer’s protocol using QIAcube connect, including in-column treatment with DNAse I treatment (Qiagen, Catalog 79254). The quantity and purity of obtained RNA was assessed using NanoDrop (Thermo Scientific, USA), and quality was assessed using the Agilent 4200 Tapestation using RNA screen tape (Agilent, Santa Clara, CA, Catalog 5067-5576). RNA samples having RIN value > 6 were sent to SeqCenter (Pittsburgh, PA, USA) for sequencing. At SeqCenter, total RNA samples were treated with DNase I (Invitrogen) to remove the genomic DNA, and ribosomal RNA was removed using Ribo Zero Plus rRNA Depletion kit (ILLUMINA, Catalog 20037135). Thus, obtained rRNA-depleted RNA samples were further used for cDNA synthesis and library preparation using Illumina Stranded Total RNA Prep Kit (Catalog 20040,25). The obtained library was sequenced using the Illumina Novaseq platform to produce 2 × 150 base pair read.

### Bioinformatics and statistical analysis

Raw reads obtained from the Illumina platform were subjected to quality filter and adapter trimming by the BCL converter tool (v4.2.4). Quality of raw reads was further assessed using FastQC (0.12.1). Since rRNA removal was done in wet-lab stage prior to sequencing, no additional computational rRNA filtering was performed. Filtered reads were aligned to reference *Salmonella* Infantis genome using HISAT2 (2.2.1) (25–27). Aligned readings were processed using HTSeq (2.0.9) to generate gene-level counts matrices (28). The differential expression of genes was analyzed using DESeq2 package in R v. 4.4.3 (29). Size factors were estimated using only *Salmonella*-mapped gene counts such that unmapped non-*Salmonella* reads did not contribute to normalization or variance estimation. To account for the multiple testing, *P*-values were adjusted using the Benjamini-Hochberg procedure, and a significance threshold of FDR < 0.05 and a |log2FoldChange| ≥ 2 was applied. We maintained the default DESeq2 settings for independent filtering of low-count transcripts and the Cook’s distance cutoff for outlier detection to ensure the robustness of the identified differentially expressed genes (DEGs). Pairwise, hypothesis-driven comparisons were conducted for three biologically relevant treatments contrasts: BHI vs PBS, BHI vs BHI+Litter, and PBS vs PBS+Litter.

## RESULTS AND DISCUSSION

### Alignment with reference genome

On average, approximately 50 million paired reads were obtained per sample. When bacteria were cultured in BHI and PBS, there were high degrees of resemblance (approximately 90%) to the reference genome. Conversely, the average bacterial gene alignment to reference genomes was reduced to approximately 50% when *S*. Infantis were cultured with litter in BHI and PBS. The reduction of the *S*. Infantis when it was cultured in litter might be because of the interference from the background bacterial flora that was already present in the litter (Table 2; Tables S1 and S2).

**TABLE 2:** Average alignment of RNA sequences with *Salmonella* Infantis reference genome

SN	Treatment	Average alignment (%)
1	BHI+Litter	52.54
2	PBS+Litter	49.81
3	BHI	93.15
4	PBS	88.1

**Fig 1:**
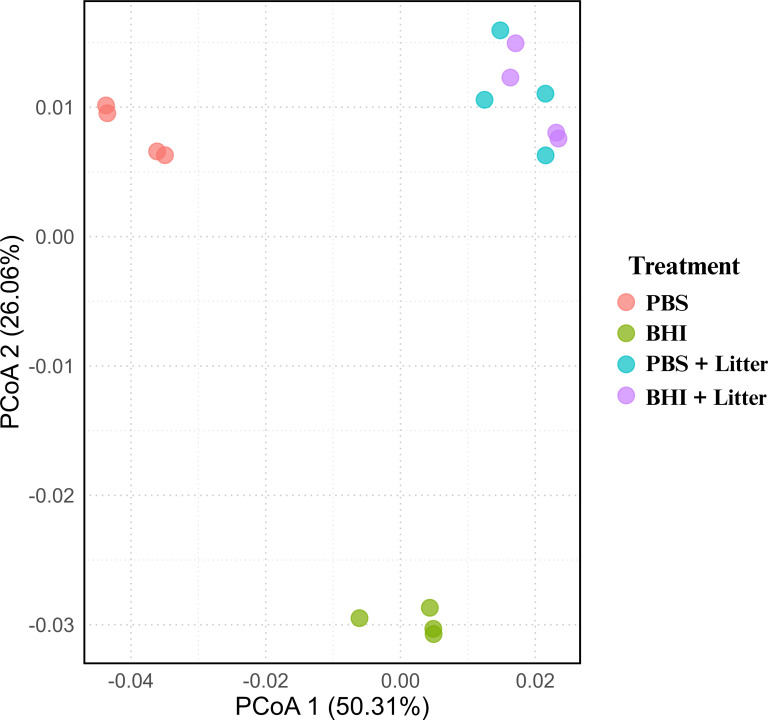
Principal coordinate analysis (PCoA) illustrating the transcriptomic differences across four treatment groups. The plot visualizes the differences in gene expression profiles for *Salmonella* Infantis grown in nutrient-rich (BHI) and nutrient-poor (PBS) conditions, both in the presence and absence of litter. The ordination is based on *a* Bray-Curtis dissimilarity matrix calculated from variance stabilizing transformed (VST) gene counts. The first principal coordinate axis (PCoA1) captures 50.31% of the total variation, and the second axis (PCoA2) captures 26.06%.

The principal component analysis (PCoA) revealed that treatment replicates were clustered closely as shown in Fig. 1. The treatments PBS and BHI formed distinct cluster, whereas PBS+Litter and BHI+Litter formed almost similar clusters (Fig. 1). While comparing differently expressed genes between the BHI and BHI+Litter, a total of 187 genes were upregulated and 284 downregulated. Similarly, a total of 391 genes were upregulated, and 341 genes downregulated between the PBS in comparison to PBS+Litter. A total of 406 genes were upregulated and 314 downregulated in PBS compared to BHI (Table 3; Fig. S1 to S3).

**TABLE 3:** Differentially expressed *Salmonella* Infantis genes across treatments^*a*^

Treatment	Gene counts	Median |log2FC|
PBS+Litter vs PBS		
Up in PBS+Litter genes	386	2.76
Down in PBS+Litter genes	332	2.75
Total significant genes	718	
BHI+Litter vs BHI		
Up in BHI+Litter genes	171	2.46
Down in BHI+Litter genes	289	2.68
Total significant genes	460	
PBS vs BHI		
Up in PBS genes	307	2.44
Down in PBS genes	397	2.77
Total significant genes	704	

^*a*^ The table summarizes the total number of significantly upregulated and downregulated genes in *Salmonella* Infantis. FDR ≤ 0.05.

### Differentially expressed genes

#### Starvation stress

Nutrient availability is important for survival and replication; however, scarcity of nutrients allows bacteria to develop several survival mechanisms that enable them to persist in the surroundings (30). The *rpoS* gene provides protection to *Salmonella* against various stress factors like deficiency of nutrients, oxidative stress, changes in temperature and pH, and regulation of virulent factors (31). The upregulation of the *rpoS* gene in PBS in comparison to PBS+Litter suggests the development of a survival strategy in nutrient-deficient conditions like PBS (Table 4; Fig. S4). The *dadA* gene is associated with catalyzation of the oxidative deamination of D-amino acids like alanine in *Salmonella* and *E. coli* (32). Conversely, the *dadA* gene was downregulated in PBS in comparison to PBS+Litter (Table 4; Fig. S4). The increased expression of *dadA* in litter in comparison to PBS suggests that *Salmonella* could utilize amino acids from the organic material available in litter as the nutrient material for survival when the nutrient is not readily available.

**TABLE 4:** Differential expressions of *Salmonella* starvation stress genes under different culture conditions^*a*^

Starvation stress gene	Base mean	BHI+Litter vs BHI (log2FC)	PBS+Litter vs PBS (log2FC)	PBS vs BHI (log2FC)
*rpoS*	168,248.5	–	−2.21	–
*eutS*	139.5	–	−2.33	2.70
*pduF*	484.11	–	−2.00	–
*fadD*	12,073.72	−2.13	−3.95	2.07
*fadE*	175,857.5	−2.58	−4.90	2.56
*dksA*	6,644.63	–	−3.02	–
*fadB*	18,548.08	−2.65	−4.33	–
*fadA*	8,458.346	−2.54	−4.21	–
*fadH*	4,906.19	−2.39	–	–
*fadI*	5,414.05	−2.67	−2.77	–
*fadL*	9,514.197	−3.36	−2.93	–

^*a*^ The table shows the log2fold change (log2FC) values for the selected starvation stress-associated genes in *Salmonella* Infantis when grown under three comparative conditions. Base Mean indicates the average normalized expression across all samples. Positive values indicate upregulation and negative values indicate downregulation in the first condition relative to the second (e.g., −2.13 for *fadD* in BHI+Litter vs BHI indicates lower expression in BHI+Litter). A dash (–) denotes no significant change (FDR > 0.05) in expression under the corresponding condition.

The *dksA* gene plays an important role in regulating metabolic pathways so that bacteria can survive in minimal nutrient conditions (33). The *dksA* gene was upregulated in bacteria grown in PBS compared to PBS+Litter and BHI (Table 4; Fig. S4), which indicates that the *dksA* gene might have played an important role in bacterial survival during starvation by controlling the metabolic function. Ethanolamine (*eut*) and propanediol (*pdu*) operons encode for genes that enable *Salmonella enterica* to utilize ethanolamine and 1,2-propanediol, respectively, as a source of energy for several metabolic pathways (34). The upregulation of the *eutS* and *pduF* genes in *S*. Infantis when cultured in PBS compared to PBS+Litter indicates bacteria in PBS might be attempting to utilize alternative energy sources like ethanolamine and propanediol for survival and growth during starvation (35).

Fatty acid degradation (*fad*) genes such as *fadA*, *fadB*, *fadD*, *fadE*, *fadI*, and *fadL* were downregulated in BHI+Litter and PBS+Litter in comparison to BHI and PBS, respectively (Table 4; Fig. S4). Furthermore, the downregulation of *fad* genes in bacteria cultured in litter indicates that the bacteria had sufficient nutrient materials for survival and growth, so bacteria were not using fatty acid derivatives to produce energy. However, the *fad* genes were upregulated in PBS and BHI in comparison to PBS+Litter and BHI+Litter, respectively, suggesting the utilization of fatty acids such as phospholipids available in the cell membrane of the *S*. Infantis for survival (36). In addition, an upregulation of *fadD* and *fadE* was observed in PBS in comparison to BHI in this study. This might occur in response to the nutrient scarcity in PBS, enabling *S*. Infantis to utilize the fatty acids in their cell membrane for survival. Collectively, *S*. Infantis demonstrated the diverse utilization of environmental substrates as nutrients to derive energy and ensure survival in scarce nutrient conditions.

#### Expression of alternative (anaerobic) respiration systems

Bacteria extract electrons from energy-rich substrates and transport them through the electron transport chain for ATP synthesis, which is crucial for growth and multiplication (37). There are diverse alternative respiratory pathways utilized by bacteria for energy generation by ATP synthesis. Respiratory formate dehydrogenases-N (*fdnGH*) genes convert the formate into carbon dioxide and pass electrons for reduction to anaerobic respiratory substrates like nitrate, nitrite, trimethylamine *N*-oxide, dimethyl sulfoxide, and fumarate in anaerobic conditions (38). The *fdnG* and *fdnH* genes were also found to be upregulated in PBS+Litter and BHI in comparison to PBS (Table 5; Fig. S5), which indicates that bacteria might be switching to an alternative respiratory pathway to utilize nutrients available in litter for survival in oxygen-deficient conditions (39).

**TABLE 5:** Differential expressions of *Salmonella* anaerobic respiration genes under different culture conditions^*a*^

Anaerobic respiration gene	Base mean	BHI+Litter vs BHI (log2FC)	PBS+Litter vs PBS (log2FC)	PBS vs BHI (log2FC)
*fdnH*	9,233.14	–	5.34	−6.29
*fdnG*	871.52	–	2.59	−2.25
*dmsA*	2,035.24	–	3.07	−2.82
*dmsB*	462.71	–	3.35	−3.50
*dmsC*	276.86	–	2.62	−2.72
*dmsD*	1,388.89	–	–	−2.76
*ttrC*	569.24	–	2.76	−2.51
*ttrB*	899.78	–	3.25	−2.25
*ttrS*	3,587.88	−2.01	–	–

^*a*^ The table shows the log2fold change (log2FC) values for the selected anaerobic respiration-associated genes in *Salmonella* Infantis when grown under three comparative conditions. Base Mean indicates the average normalized expression across all samples. Positive values indicate upregulation, and negative values indicate downregulation in the first condition relative to the second (e.g., 5.34 for *fdnH* in PBS+Litter vs PBS indicates higher expression in PBS+Litter). A dash (–) denotes no significant change (FDR > 0.05) in expression under the corresponding condition.

The *dmsA, dmsB,* and *dmsC* genes were found to reduce dimethyl sulfoxide (DMSO) to dimethyl sulfide in anaerobic conditions during oxidative stress for energy generation to ensure survival of bacteria (40). An upregulation of *dmsA, dmsB,* and *dmsC* genes was observed in PBS+Litter and BHI in comparison to PBS (Table 5; Fig. S5; |log₂FC| ≥ 2, FDR ≤ 0.05). This result indicates that *Salmonella* could activate the DMSO reductase system under these unfavorable conditions such as presence of litter. Additionally, *ttrS* is a sensor protein in a two-component regulatory system to detect environmental signals to activate tetrathionate respiration, and *ttrB* and *tttC* genes utilize tetrathionate for respiration under anaerobic conditions (36). The *ttrB* and *ttrC* genes were found to be upregulated in PBS+Litter and BHI in comparison to PBS in this study (Table 5; Fig. S5). This suggests that *S*. Infantis is utilizing tetrathionate for energy generation. To summarize, it was found that *S*. Infantis can regulate the gene expression for an alternative respiratory pathway depending upon the energy sources available in the medium to ensure survival.

#### Oxidative stress genes

Reactive oxygen species (ROS) are the intermediates, such as hydrogen peroxide, hydroxyl radicals, and superoxide anion radicals, that are produced during bacterial respiration (41, 42). The ROS are highly reactive, and they degrade the cellular components of bacterial cells (43). *Salmonella* has developed several defense mechanisms to prevent damage from such ROS intermediates (44). The *arcA* gene is associated with anaerobic stress in *Salmonella* that modulates the pathway of metabolism, flagellar biosynthesis, and motility (45). The *arcA* gene was downregulated in BHI+Litter in comparison to BHI but upregulated in BHI in comparison to PBS (Table 6; Fig. S6). The downregulation of the *arcA* gene in BHI+Litter in comparison to BHI might be due to the presence of microflora in litter that could eliminate *S*. Infantis through competitive exclusion, which is absent from bacteria in BHI in comparison to PBS. Catalase-peroxidase gene (*katG*) is the gene regulated by oxyR regulon in *Salmonella* that degrades peroxides to water and molecular oxygen (46). Alkyl hydroperoxide reductase gene (*ahpC*) is another gene regulated by the oxyR regulon that converts organic hydrogen peroxides to alcohols (44). An upregulation of *ahpF* and *ahpC* genes was observed in BHI+Litter and PBS+Litter in comparison to BHI and PBS, respectively (Table 6; Fig. S6). In addition, the *katG* gene was also upregulated in PBS+Litter in comparison to PBS. *soxRS* is a pair of regulatory genes in *E. coli* where the s*oxR* gene acts as a sensor for ROS and on oxidation, the *soxR* gene activates the *soxS* gene to produce several proteins that defend against oxidative stressors (47). The *soxR* and *soxS* genes were upregulated in BHI+Litter in comparison to BHI, but *soxR* and *soxS* were downregulated in BHI in comparison to PBS. The upregulation of the *katG, ahpF*, *ahpC,* and *soxS* genes in this study might suggest that oxidative stress was developed during growth, and *S*. Infantis is activating the defense mechanism for survival (Table 6; Fig. S6). Heat stress occurs in bacteria when there is an increase in the optimum temperature of the medium that enables bacteria to synthesize and accumulate harmful heat shock proteins that usually occur in response to an abrupt elevated temperature (48). The accumulation of such proteins is sensed by the *cpxA/cpxR* two-component bacterial transduction system, which mobilizes *cpxp* to increase the expression of various anti-stress proteins like *degP* (49). A study by Strauch et al. (50) revealed that *degP* genes regulate the secretion of a cell membrane protease that lyses the harmful proteins accumulated in the outer membrane of *E. coli* (50). In this study, *cpxp* and *degP* genes were downregulated in BHI+Litter and PBS+Litter in comparison to BHI and PBS, respectively (Table 6; Fig. S6), which implies that *S*. Infantis was incubated at optimum temperature and was not exposed to any heat stress. Furthermore, the downregulation of *cpxP* and *degP* genes in the *S*. Infantis incubated litter might also be due to the acidic environment of the litter which is similar to the findings by Danese and Silhavy (51) which revealed that *cpxP* transcription is strongly induced in alkaline conditions in comparison to acidic environment. Altogether, this study found that the generation of free radicals during the utilization of nutrients for growth and multiplication by *S*. Infantis was neutralized by synthesizing enzymes that degrade the ROS intermediates.

**TABLE 6:** Differential expressions of *Salmonella* oxidative stress genes under different culture conditions^*a*^

Oxidative stress gene	Base mean	BHI+Litter vs BHI (log2FC)	PBS+Litter vs PBS (log2FC)	PBS vs BHI (log2FC)
*arcA*	72.28	−3.08	–	−2.79
*ahpF*	16,304.31	4.19	3.22	–
*ahpC*	61,950.78	3.08	3.85	–
*degP*	15,312.68	−2.21	−2.29	–
*soxR*	883.47	2.37	–	3.87
*soxS*	346.66	2.46	–	2.86
*katG*	23,034.18	–	2.62	−2.19
*cpxP*	177,311.9	−3.45	−7.54	4.76

^*a*^ The table shows the log2fold change (log2FC) values for the selected oxidative stress-associated genes in *Salmonella* Infantis when grown under three comparative conditions. Base Mean indicates the average normalized expression across all samples. Positive values indicate upregulation and negative values indicate downregulation in the first condition relative to the second (e.g., −3.08 for *arcA* in BHI+Litter vs BHI indicates lower expression in BHI+Litter). A dash (–) denotes no significant change (FDR > 0.05) in expression under the corresponding condition.

#### Acid and osmotic stress

Multiple acid tolerance mechanisms are developed in *Salmonella* to resist the acidic environment in the gut (52). The lysine decarboxylase (*cadA*) gene and the lysine-cadaverine antiporter (*cadB*) genes were found to help in regulating acid stress in *S*. Typhimurium and arginine decarboxylase (*adiA*) gene regulated acid stress in *E. coli* (53). In this study, amino acid-dependent acid resistance mechanism genes such as *adiA, cadA*, and *cadB* were found to be upregulated in BHI+Litter in comparison to BHI (Table 7; Fig. S7). This indicates that acid stress might have developed in the medium with litter, which was regulated by the acid stress genes to reduce the acid stress. Anaerobic sulfite reduction genes *asrA, asrB,* and *asrC* were upregulated in BHI+Litter and PBS+Litter in comparison to BHI and PBS, respectively (Table 7; Fig. S7). The upregulation of *asrA, asrB,* and *asrC* genes in litter might be due to the development of an acidic environment resulting from the breakdown of organic materials during bacterial multiplication and growth, which is similar to the finding that *asr* genes were found to be induced in response to an acidic environment in *E. coli* (54). Overall, it can be inferred that acid stress could have developed while utilizing the nutrients by the *S*. Infantis, especially in litter, which was regulated by the upregulation of various acid stress resistance mechanisms by the bacteria in this study. Additionally, in the study, the *osmW, osmX,* and *osmY* genes were upregulated in PBS+Litter and BHI in comparison to PBS (Table 7; Fig. S7). Upregulation suggests the survival strategy of bacteria to manage osmotic stress that could have generated during nutrient utilization by *S*. Infantis as these genes were found to be active during adverse environmental conditions such as hyperosmolarity, growth phase signals, and antibacterial peptide stress for adaptation (55).

**TABLE 7:** Differential expressions of *Salmonella* acid and osmotic stress genes under different culture conditions^*a*^

Acid and osmotic stress gene	Base mean	BHI+Litter vs BHI (log2FC)	PBS+Litter vs PBS (log2FC)	PBS vs BHI (log2FC)
*adiA*	1,811.27	2.30	–	–
*asrA*	1,552.25	3.55	4.12	–
*asrB*	1,083.31	3.13	3.22	–
*asrC*	2,138.21	2.63	2.49	–
*cadA*	2,983.23	3.90	–	–
*cadB*	2,453.30	4.54	–	–
*osmB*	1,078.31	–	–	2.09
*osmW*	1,445.45	–	2.68	−2.49
*osmX*	2,187.10	–	2.37	−2.30
*osmY*	1,521.07	–	2.33	−2.37

^*a*^ The table shows the log2fold change (log2FC) values for the selected acid and osmotic stress-associated genes in *Salmonella* Infantis when grown under three comparative conditions. Base Mean indicates the average normalized expression across all samples. Positive values indicate upregulation and negative values indicate downregulation in the first condition relative to the second (e.g., 2.30 for *adiA* in BHI+Litter vs BHI indicates higher expression in BHI+Litter). A dash (–) denotes no significant change (FDR > 0.05) in expression under the corresponding condition.

#### Antimicrobial peptide stress

Antimicrobial peptides (AMPs) are the peptide molecules innately secreted by animals and plants that function to inhibit the entry of invading pathogens into the host (56). On encountering the antimicrobial peptides, pathogenic bacteria can regulate their gene expression for survival and virulence (57). The *ygiC* gene is associated with enzyme glutathionylspermidine (GSP) synthetases that synthesize glutathionylspermidine and reduce toxic metabolites from amino acid derivatives (58). The downregulation of the *ygiC* gene in BHI+Litter in comparison to BHI observed in this study indicates that abundant nutrients in the medium BHI+Litter reduced the need of *S*. Infantis to utilize the amino acid derivatives for nutrients (Table 8; Fig. S8). As a result, there was less generation of amino acid-related metabolites in BHI+Litter which might have caused downregulation of the *ygiC* gene in BHI+Litter. PhoP-activated gene (*pagN*) is a gene regulated by the PhoPQ two-component transcription regulatory system in *S*. Typhimurium (59). The deletion of the *pagN* gene *in vitro* mammalian cell lines resulted in a reduction in virulence due to poor adhesion and invasion by *S*. Typhimurium (60). In this study, the *pagN* gene was downregulated in PBS+Litter in comparison to PBS, which might be a mechanism for survival for *Salmonella* since there is high bacterial competition in the litter. The Rcsc protein is a transmembrane sensor kinase which is a part of the rcsCDB phosphorelay system that senses bacterial cell envelope stress and regulates cell membrane structures like polysaccharides, flagella, and fimbriae in response to the host’s environmental condition, thus regulating virulence in Enterobacteriaceae (61, 62). The *rcsC* gene was downregulated in BHI+Litter and PBS+Litter in comparison to BHI and PBS while it was upregulated in BHI as compared to PBS. The downregulation of *rcsc* genes in litter suggests the litter environment might have microbial competition where the *S*. Infantis is reducing the capsule synthesis and ensuring survival. The *marRAB* operon in *S*. Typhimurium consists of *marA* and *marR* genes, where the *marA* gene promotes multidrug resistance by activating several resistance-related genes, while *marR* suppresses the operon under normal conditions (63). The genes *marA* and *marR* were upregulated in BHI+Litter and PBS+Litter in comparison to BHI and PBS, respectively, but the *marR* gene was downregulated in BHI in comparison to PBS in this study (Table 8; Fig. S8). This indicates that *S*. Infantis is likely activating its defense due to the exposure of antimicrobial molecules likely present in the litter medium. Altogether, several mechanisms were found to be utilized by the bacteria for their survival in different mediums.

**TABLE 8:** Differential expressions of *Salmonella* antimicrobial peptide genes under different culture conditions^*a*^

Antimicrobial peptide gene	Base mean	BHI+Litter vs BHI (log2FC)	PBS+Litter vs PBS (log2FC)	PBS vs BHI (log2FC)
*marA*	11,868.43	6.48	3.95	–
*marR*	10,412.71	6.25	3.14	2.08
*pagN*	353.60	–	−2.24	–
*rcsC*	4,941.67	−4.78	−2.18	−2.22
*ygiC*	10,862.72	−2.22	–	–

^*a*^ The table shows the log2fold change (log2FC) values for the selected antimicrobial peptides-associated genes in *Salmonella* Infantis when grown under three comparative conditions. Base Mean indicates the average normalized expression across all samples. Positive values indicate upregulation and negative values indicate downregulation in the first condition relative to the second (e.g., 6.48 for *marA* in BHI+Litter vs BHI indicates higher expression in BHI+Litter). A dash (–) denotes no significant change (FDR > 0.05) in expression under the corresponding condition.

#### Cell envelope structures, chemotaxis, and motility

The cell envelope in *Salmonella* is an important cell constituent that enables protection from the host cellular environment, regulates the uptake of nutrients, and restricts the movement of harmful substances (64). The cell membrane plays a crucial role in bacterial survival at times of nutrient deficiency and immune response. The *yohc* gene is a putative transport gene that is activated during the carbon starvation stress response in *Salmonella* Typhimurium (65). The *yohC* gene was upregulated in PBS in comparison to PBS+Litter and BHI. The upregulation of *yohC* genes in nutrient-poor conditions like PBS in comparison to nutrient-rich conditions like PBS+Litter and BHI in this study suggests the bacterial adaptation response to starvation and survival stress (Table 9; Fig. S9). The *yddg* gene encodes a protein for the export of small aromatic amino acid molecules out of the bacterial cell to maintain metabolic balance and cellular homeostasis in *E. coli* (66). An upregulation of the *yddG* gene was observed in PBS+Litter and BHI in comparison to PBS in the study. This indicates that *S*. Infantis is actively removing accumulated amino acid products to ensure survival in PBS+Litter and BHI in comparison to PBS. The *omp* genes encode for outer membrane proteins that form porins (pores) in the cell membrane of *Salmonella,* which play a crucial role in the transport of nutrients, virulence, and antibiotic resistance (67, 68). The genes *ompC, ompD, ompF,* and *ompW* regulate the movement of ions through the porin channels in the outer membrane of *Salmonella* in response to temperature, composition, and osmolarity of growth media in gram negative bacteria (69, 70). *The ompD* gene was found to be downregulated in BHI+Litter in comparison to BHI in this study. On the other hand, *ompD* genes were upregulated in PBS+Litter and BHI in comparison to PBS. The downregulation in BHI+Litter in comparison to others might be due to an increase in the acidity of this medium. A similar observation was made by Santiviago et al. (71) who also observed a reduced expression of the *ompD* gene in *S*. Typhimurium when grown in LB medium at acidic vs neutral conditions. The *ompW* gene encodes for a porin that was found to efflux the methyl viologen out of *S*. Typhimurium in an experimental study (67). In this study, *ompW* was also upregulated in PBS+Litter and BHI in comparison to PBS. The upregulation of the *ompW* gene might be a survival mechanism of *S*. Infantis to remove harmful molecules in conjunction with the *ompD* gene to maintain osmolarity in the medium. The *ompF* gene directs the transport of nutrients and ions in the bacteria while removing waste, including efflux of antibiotics (72). The downregulation of *ompF* genes in BHI+Litter and PBS+Litter in comparison to BHI and PBS, respectively, was found in this study. The downregulation might be a survival strategy by *S*. Infantis to suppress their presence in litter microflora to avoid competitive exclusion. The *ompX* is another gene encoding a minor porin protein that responds to oxidative (H_2_O_2_) stress, host invasion, iron homeostasis, and recognition of bacteria by the host’s adaptive immune response (73). The downregulation of *ompX* was observed in PBS+Litter and BHI in comparison to PBS. The significance of *ompX* genes was not understood in this study. The *ompF* gene directs the transport of nutrients and ions in the bacteria while removing waste, including efflux of antibiotics (72). The downregulation of *ompF* genes in BHI+Litter and PBS+Litter in comparison to BHI and PBS, respectively, was found in this study. This downregulation might be a survival strategy by *S*. Infantis to suppress its presence within the litter microflora to avoid competitive exclusion.

**TABLE 9:** Differential expressions of *Salmonella* cell envelope, chemotaxis, and motility genes under different culture conditions^*a*^

Cell envelope, chemotaxis, and motility gene	Base mean	BHI+Litter vs BHI (log2FC)	PBS+Litter vs PBS (log2FC)	PBS vs BHI (log2FC)
*cheA*	1,655.41	−2.05	–	−2.31
*cheB*	937.53	−2.98	2.85	−4.44
*cheR*	953.66	−3.26	2.39	−4.38
*cheY*	358.54	−2.67	–	−3.11
*cheZ*	1,000.06	−2.32	–	−2.46
*fliA*	1,263.79	–	4.72	−3.92
*fliC*	33,151.26	−2.54	3.37	−3.35
*fliD*	3,153.21	−2.40	3.69	−4.26
*fliE*	157.38	–	2.24	–
*fliF*	723.46	–	2.53	–
*fliG*	346.28	–	2.071	-
*fliH*	337.33	–	2.31	–
*fliI*	316.12	–	2.88	–
*fliJ*	150	–	2.25	–
*fliK*	664.94	–	2.75	–
*fliM*	546.05	–	2.57	–
*fliN*	119	–	2.09	–
*fliO*	90.75	–	2.12	–
*fliS*	343.77	−2.18	2.22	−3.07
*fliT*	277.34	–	2.21	−2.87
*fliY*	6,958.64	−2.23	3.36	2.11
*fliZ*	568.35	–	4.72	−2.20
*ompC*	1,151.66	–	−2.57	4.30
*ompD*	65,013.27	−2.68	4.71	−6.43
*ompF*	9,629.82	−3.39	−2.07	2.02
*ompW*	81,552.45	–	−2.17	−6.53
*ompX*	84,003.95	–	5.69	2.41
*yddG*	330.77	–	2.93	−2.70
*yohC*	28,218.56	–	−5.50	4.78

^*a*^ The table shows the log2fold change (log2FC) values for the selected cell envelopes, chemotaxis, and motility-associated genes in *Salmonella* Infantis when grown under three comparative conditions. Base Mean indicates the average normalized expression across all samples. Positive values indicate upregulation and negative values indicate downregulation in the first condition relative to the second (e.g., −2.05 for *cheA* in BHI+Litter vs BHI indicates lower expression in BHI+Litter). A dash (–) denotes no significant change (FDR > 0.05) in expression under the corresponding condition.

Flagella is a long filamentous structure in the outer membrane of *Salmonella* with distinct morphological assembly of basal body, hook, and filament, and it helps in locomotion as well as invading the enterocytes following oral inoculation in the host (74). The synthesis of structural components of motile and functional flagella depends upon the interaction of several genes in bacteria that regulate the first, second, and third stages of flagellar apparatus synthesis (75, 76). Genes like *fliA, fliC, fliD, fliE, fliF, fliG, fliH, fliJ, fliK, fliL, fliM, fliN, fliO, fliS, fliT,* and *fliZ* were upregulated in PBS+Litter in comparison to PBS, while only the *fliO* gene was upregulated in BHI+Litter in comparison to BHI (Table 9; Fig. S9). The upregulation of several *fli* genes in PBS+Litter indicates the development of flagella in *S*. Infantis in the medium PBS+Litter in comparison to PBS (77). This upregulation might be to utilize the nutrients available in the medium PBS+Litter, which may not be necessary for the bacteria due to the abundant nutrients in the BHI+Litter medium. *flhD, fliA, fliC, fliD, fliS, fliT,* and *fliZ* genes were also found to be upregulated in BHI in comparison to PBS. The downregulation of *fli* genes in PBS in comparison to BHI might be for energy conservation to increase the survival of *S*. Infantis by reducing the energy expenditure in flagellum synthesis. The *flhD* gene is a part of the master operon *flhCD* that dictates the synthesis of flagella in *Salmonella* (76). The upregulation of *flhD* by *S. Infantis* in BHI in comparison to PBS indicates the development of the flagellar apparatus to ensure motility. Minamino et al. (78) report that *fliB* and *fliC* genes are two distinct genes that are involved in the synthesis of flagellin filament in *S. enterica*. The upregulation of flagellar genes in BHI in comparison to PBS might be due to the availability of nutrients for bacterial proliferation. In addition, *Salmonella* has receptors in the cell surface that relay environmental signals to flagella to induce chemotaxis (79). In this study, chemotaxis genes *cheA, cheB, cheR, cheY,* and *cheZ* were found to be upregulated in PBS+Litter and BHI in comparison to PBS, but *cheB, cheR, cheY,* and *cheZ* were downregulated in BHI+Litter in comparison to BHI (Table 9; Fig. S9). *S*. Infantis likely have assessed nutrient availability and environmental conditions utilizing the *che* genes which have been described as an innate behavior of bacteria to increase survival (80). The downregulation of chemotaxis and flagellar apparatus genes in BHI+Litter in comparison to BHI might be a concurrent development in *S*. Infantis to ensure survival, which is in agreement with the report made by Stock et al. (81) that found *cheA* and *cheY* regulate chemotaxis as an adaptive response to changing environments in *E. coli* and *S*. Typhimurium. It could be interpreted that chemotaxis was utilized by *S*. Infantis to search for nutrients in PBS+Litter by developing flagella, which was not observed in BHI+Litter. Altogether, *S*. Infantis was found to illustrate diverse mechanisms of adaptation in their outer covering in different environments to ensure their survival.

#### Virulence genes

The capability of *Salmonella* to infect the host and cause disease is dependent upon the genes clustered in the chromosome called Salmonella pathogenicity islands (SPI) (82). *Salmonella* can adapt to the changing environmental factors rapidly in order to ensure survival, which also regulates virulence (83). In this study, *hilA, invA, invF,* and *sopB* were upregulated in BHI in comparison to PBS. The gene *sopB* was also upregulated in BHI in comparison to BHI+Litter and PBS, while *sopD* and *sopE* were upregulated in BHI in comparison to BHI+Litter. In addition, *sipB, sipC,* and *sipD* were upregulated in BHI in comparison to BHI+Litter (Table 10; Fig. S10). The upregulation of *hilA* and *invF* in BHI indicates a favorable environment for the bacteria for survival and multiplication in comparison to nutrient-poor PBS. Lostroh and Lee (84) found that the activation of the *hilA* gene activates the expression of the regulatory gene *invF*. Further, *invF* in conjunction with *sicA* activates the expression of *Salmonella* invasive proteins (*sipB*) and *Salmonella* outer proteins (*sopB* and *sopE*). The increased expression of invasive proteins *sopB, sopE, sopD,* and *invA* in BHI might be due to the increased expression of *hilA* and *invF*. The introduction of the *invA* gene into non-invasive *S*. Typhimurium enabled invasiveness in tissue culture cells (85). The genes *sopB* and *sopE* help in *Salmonella* invasion into host cells through modulation of the actin cytoskeleton and membrane ruffling in host cells (86, 87), while *sopD* works concurrently with *sopB* for invasion and pathogenicity (88). In addition, the genes *sipB, sipC,* and *sipD* also facilitate the delivery of effector proteins by forming pores in the cell membrane of the host cell (89). Schechter et al. (90) found that *hilA* gene modulates gene expression in the SPI-1 secretory system in *S*. Typhimurium depending upon the environmental conditions. The competitiveness of microbes in litter might be a factor for downregulation of *hilA* and *invF* genes*,* thus resulting in reduced virulence of *S*. Infantis genes in litter. Overall, *S*. Infantis demonstrated reduced expression of virulence genes in litter in comparison to the medium without litter in our study. This suggests that bacteria adapt for survival in litter by reducing the expression of virulent genes.

**TABLE 10:** Differential expression of *Salmonella* virulence genes under different culture conditions^*a*^

Virulence gene	Base mean	BHI+Litter vs BHI (log2FC)	PBS+Litter vs PBS (log2FC)	PBS vs BHI (log2FC)
*hilA*	353.02	–	–	−2.17
*invA*	400.74	−3.19	–	−2.95
*invF*	227.05	−3.99	–	−3.01
*sicA*	266.68	−3.04		−3.30
*sicP*	45.54		2.96	−2.75
*sipB*	1,974.52	−3.71	–	−3.10
*sipD*	855.02	−2.46	–	–
*sopB*	1,184.32	−4.52		−4.31
*sopD*	316.13	−3.56	−3.57	2.07
*sopE*	113.84	−2.81	–	–

^*a*^ The table shows the log2fold change (log2FC) values for the selected virulence-associated genes in *Salmonella* Infantis when grown under three comparative conditions. Base Mean indicates the average normalized expression across all samples. Positive values indicate upregulation and negative values indicate downregulation in the first condition relative to the second (e.g., −3.19 for *invA* in BHI+Litter vs BHI indicates lower expression in BHI+Litter). A dash (–) denotes no significant change (FDR > 0.05) in expression under the corresponding condition.

### Conclusion

The survival strategies of *S*. Infantis in different mediums were observed for a period of 24 h in this study. The bacteria were found to adapt to both nutrient-rich and nutrient-scarce conditions by modulating gene expression in different sets of environmental conditions. It was revealed that the bacteria can utilize wide sets of nutrients present in the litter to derive energy for growth and multiplication using multiple routes of respiration. Also, the bacteria were found to be efficient in switching to the survival mode in accordance with the environmental stressors, such as oxidative stress, acid stress, and antimicrobial peptide stress. The expression of virulence genes of *S*. Infantis was reduced in the litter, which was an interesting observation in this study.

## Data Availability

The RNA-seq data are available under BioProject no. PRJNA1347770.

## SUPPLEMENTAL MATERIAL

Supplemental figures spectrum.03307-25-s0001.docx Fig. S1 to S10.

Supplemental tables spectrum.03307-25-s0002.xlsx Tables S1 to S6.

ASM does not own the copyrights to Supplemental Material that may be linked to, or accessed through, an article. The authors have granted ASM a non-exclusive, world-wide license to publish the Supplemental Material files. Please contact the corresponding author directly for reuse.
