# Supplementary material for: Assessing transcriptomic responses of Salmonella Infantis in the presence of poultry litter
Source: Microbiol Spectr. 2026 Mar 10;14(4):e03307-25. doi: 10.1128/spectrum.03307-25 (PMC13055217; doi:10.1128/spectrum.03307-25)
Supplement: Supplemental figures — Fig. S1 to S10. [file spectrum.03307-25-s0001.docx]

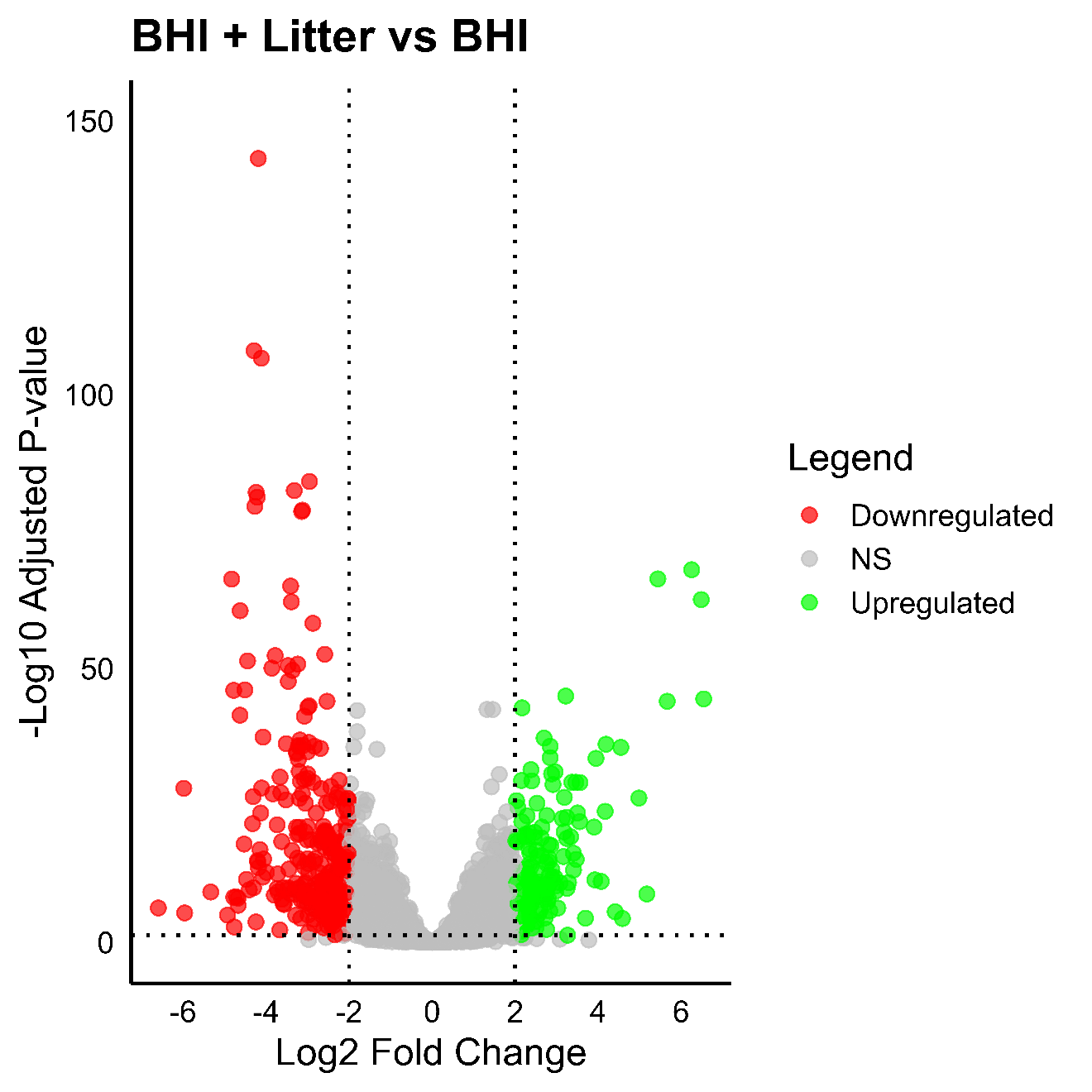


**Supplementary Figure 1**. **Volcano plot showing the differently expressed genes between BHI+Litter vs. BHI.**

RNA-seq was performed with four biological replicates per treatment, DESeq2 log₂ fold-change values were used to get upregulated and downregulated genes. Read color reflects relative down expression and green color reflects relative up regulation.


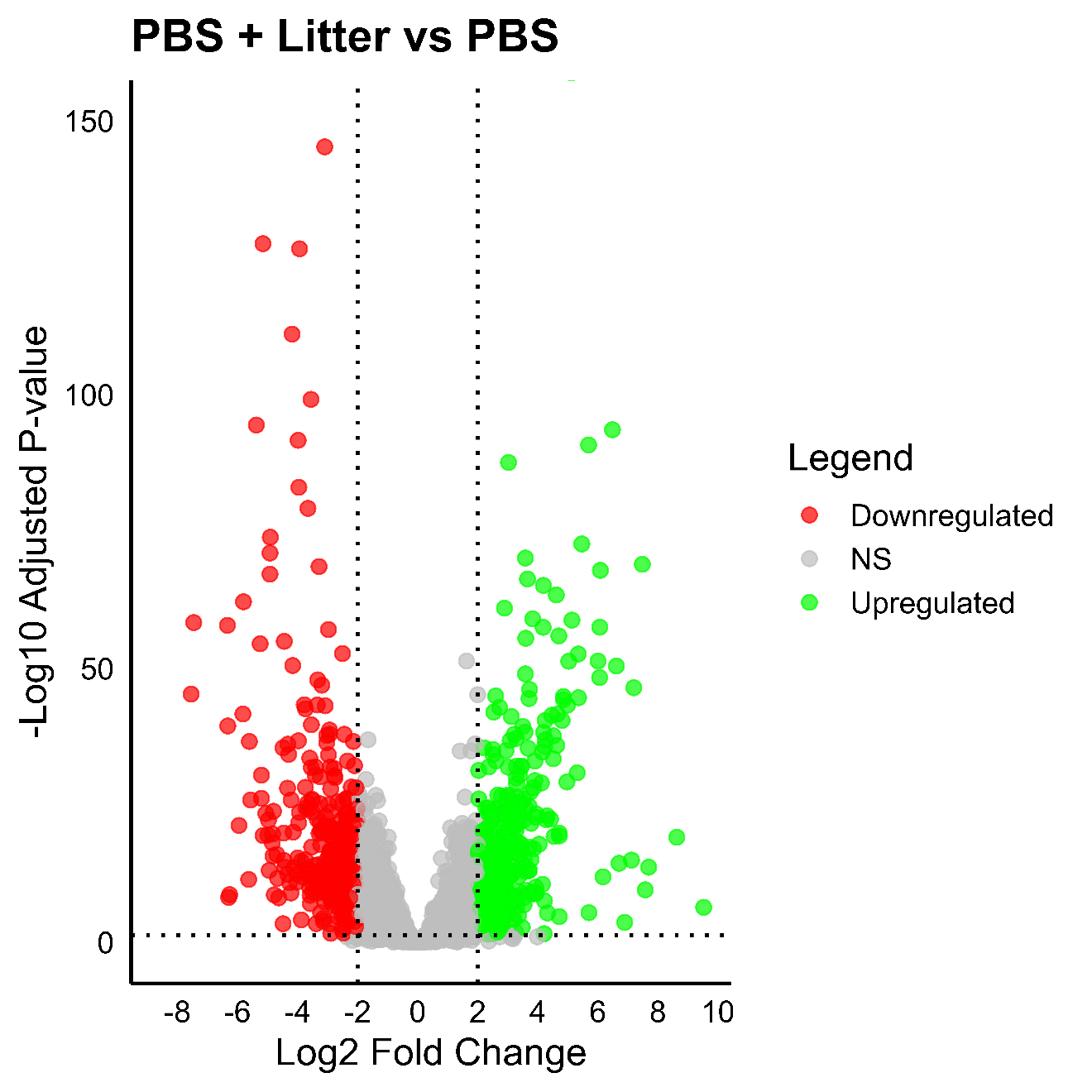


**Supplementary Figure 2**. **Volcano plot showing the differently expressed genes between PBS+Litter vs. PBS.**

RNA-seq was performed with four biological replicates per treatment, DESeq2 log₂ fold-change values were used to get upregulated and downregulated genes. Read color reflects relative down expression and green color reflects relative up regulation.


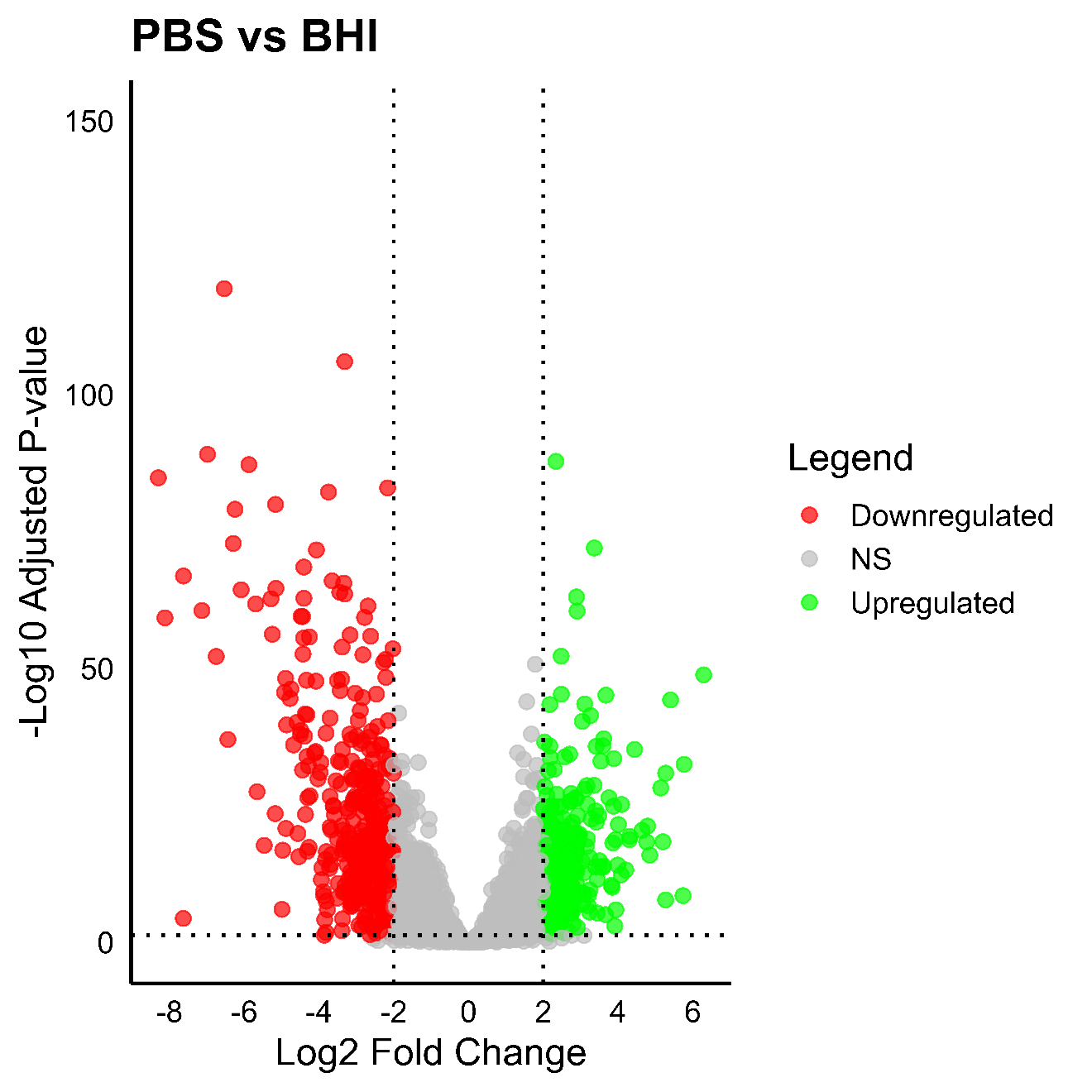


**Supplementary Figure 3**. **Volcano plot showing the differently expressed genes between PBS vs. BHI.**

RNA-seq was performed with four biological replicates per treatment, DESeq2 log₂ fold-change values were used to get upregulated and downregulated genes. Read color reflects relative down expression and green color reflects relative up regulation.


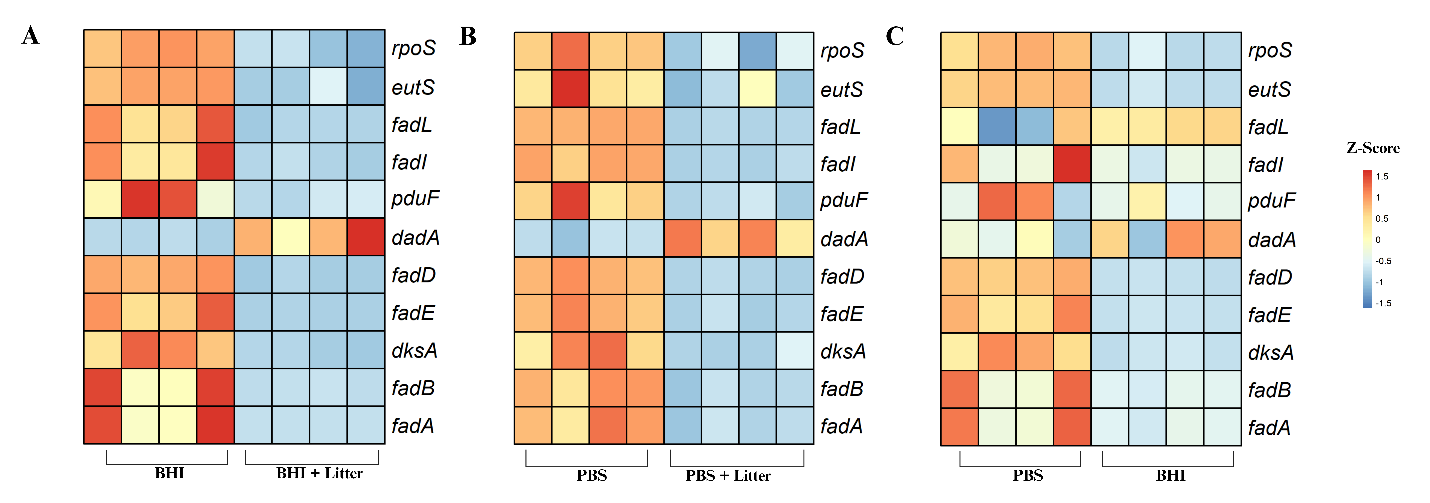


**Supplementary Figure 4**. **Heatmaps of differentially expressed genes in Salmonella Infantis across treatment conditions for starvation stress response.**

RNA-seq was performed with four biological replicates per treatment, DESeq2 log₂ fold-change values were used to get upregulated and downregulated genes. For the creation of heatmap, genes were grouped by functional categories, and heatmaps were generated using pheatmap R package with row-wise z-score scaling, so that each gene’s expression is normalized relative to its mean and standard deviation across samples. Color intensity reflects relative expression: read indicates expression above gene’s average, blue indicates expression below the gene’s average. (A) BHI vs. BHI + Litter, (B) PBS vs. PBS + Litter, and (C) PBS vs. BHI represent three pairwise comparisons made. Columns represent experimental condition, and each row represents starvation stress response genes.


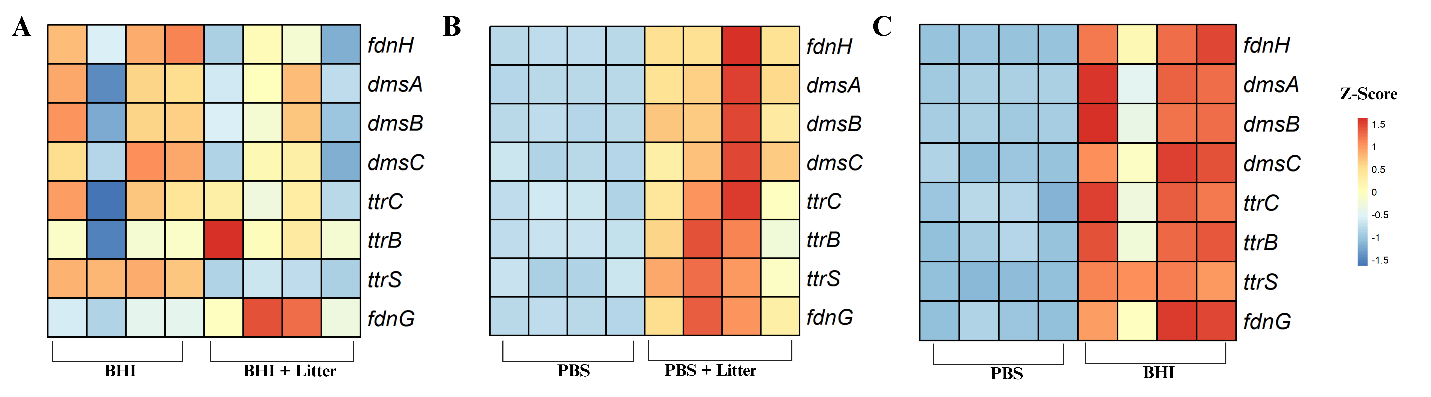


**Supplementary Figure 5**. **Heatmaps of differentially expressed genes in Salmonella Infantis across treatment conditions for anaerobic respiration genes.**

RNA-seq was performed with four biological replicates per treatment, DESeq2 log₂ fold-change values were used to get upregulated and downregulated genes. For the creation of heatmap, genes were grouped by functional categories, and heatmaps were generated using pheatmap R package with row-wise z-score scaling, so that each gene’s expression is normalized relative to its mean and standard deviation across samples. Color intensity reflects relative expression: read indicates expression above gene’s average, blue indicates expression below the gene’s average. (A) BHI vs. BHI + Litter, (B) PBS vs. PBS + Litter, and (C) PBS vs. BHI represent three pairwise comparisons made. Columns represent experimental conditions, and each row represents anaerobic respiration genes.


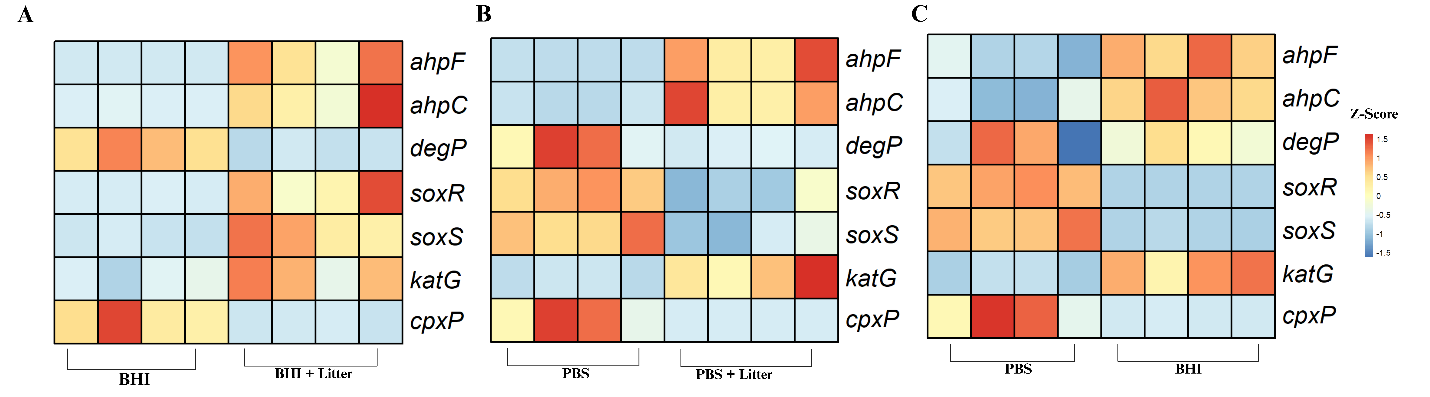


**Supplementary Figure 6**. **Heatmaps of differentially expressed genes in Salmonella Infantis across treatment conditions for oxidative stress genes.**

RNA-seq was performed with four biological replicates per treatment, DESeq2 log₂ fold-change values were used to get upregulated and downregulated genes. For the creation of heatmap, genes were grouped by functional categories, and heatmaps were generated using pheatmap R package with row-wise z-score scaling, so that each gene’s expression is normalized relative to its mean and standard deviation across samples. Color intensity reflects relative expression: read indicates expression above gene’s average, blue indicates expression below the gene’s average. (A) BHI vs. BHI + Litter, (B) PBS vs. PBS + Litter, and (C) PBS vs. BHI represent three pairwise comparisons made. Columns represent experimental conditions, and each row oxidative stress genes.


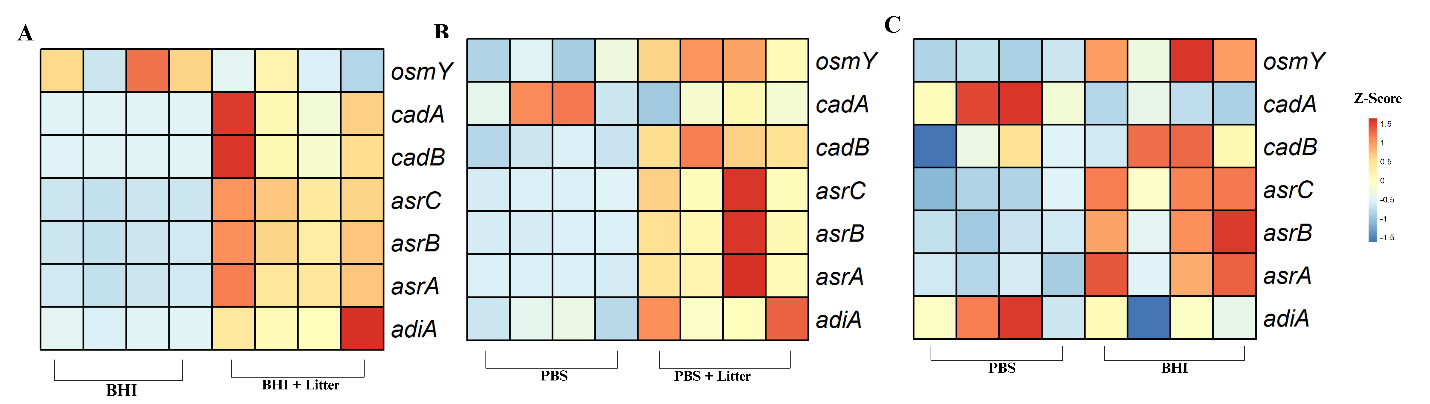


**Supplementary Figure 7**. **Heatmaps of differentially expressed genes in Salmonella Infantis across treatment conditions for acid and osmotic stress genes.**

RNA-seq was performed with four biological replicates per treatment, DESeq2 log₂ fold-change values were used to get upregulated and downregulated genes. For the creation of heatmap, genes were grouped by functional categories, and heatmaps were generated using pheatmap R package with row-wise z-score scaling, so that each gene’s expression is normalized relative to its mean and standard deviation across samples. Color intensity reflects relative expression: read indicates expression above gene’s average, blue indicates expression below the gene’s average. (A) BHI vs. BHI + Litter, (B) PBS vs. PBS + Litter, and (C) PBS vs. BHI represent three pairwise comparisons made. Columns represent experimental conditions, and each row acid stress genes.


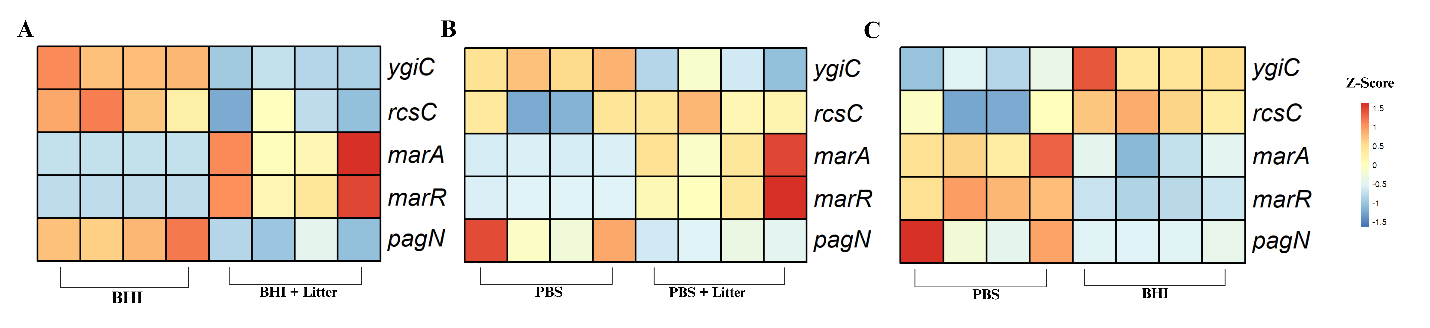


**Supplementary Figure 8**. **Heatmaps of differentially expressed genes in Salmonella Infantis across treatment conditions for antimicrobial peptides genes.**

RNA-seq was performed with four biological replicates per treatment, DESeq2 log₂ fold-change values were used to get upregulated and downregulated genes. For the creation of heatmap, genes were grouped by functional categories, and heatmaps were generated using pheatmap R package with row-wise z-score scaling, so that each gene’s expression is normalized relative to its mean and standard deviation across samples. Color intensity reflects relative expression: read indicates expression above gene’s average, blue indicates expression below the gene’s average. (A) BHI vs. BHI + Litter, (B) PBS vs. PBS + Litter, and (C) PBS vs. BHI represent three pairwise comparisons made. Columns represent experimental conditions, and each row antimicrobial peptides genes.


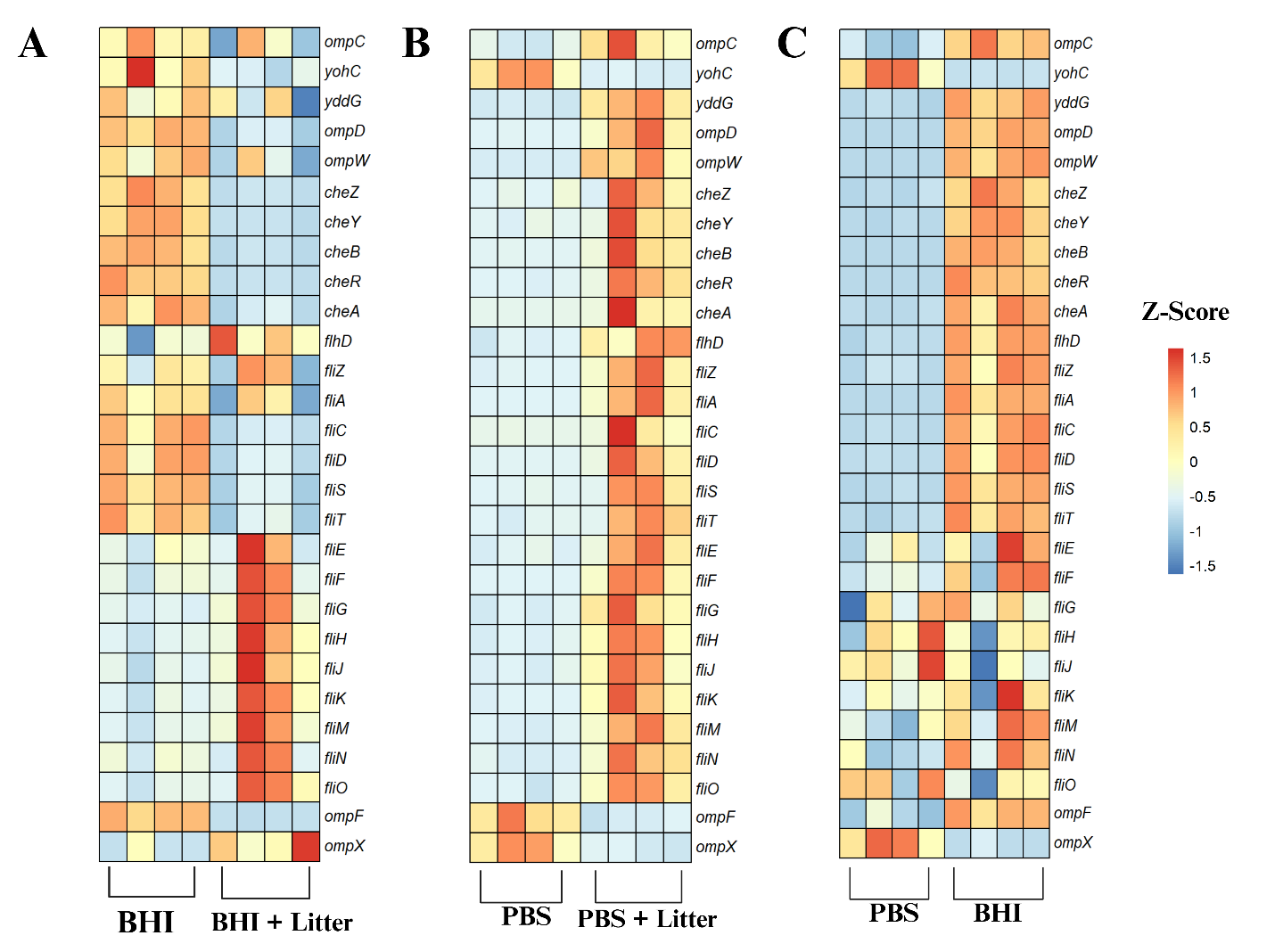


**Supplementary Figure 9**. **Heatmaps of differentially expressed genes in Salmonella Infantis across treatment conditions for cell envelopes, chemotaxis, and motility genes.**

RNA-seq was performed with four biological replicates per treatment, DESeq2 log₂ fold-change values were used to get upregulated and downregulated genes. For the creation of heatmap, genes were grouped by functional categories, and heatmaps were generated using pheatmap R package with row-wise z-score scaling, so that each gene’s expression is normalized relative to its mean and standard deviation across samples. Color intensity reflects relative expression: read indicates expression above gene’s average, blue indicates expression below the gene’s average. (A) BHI vs. BHI + Litter, (B) PBS vs. PBS + Litter, and (C) PBS vs. BHI represent three pairwise comparisons made. Columns represent experimental conditions, and each row cell envelope and motility genes.


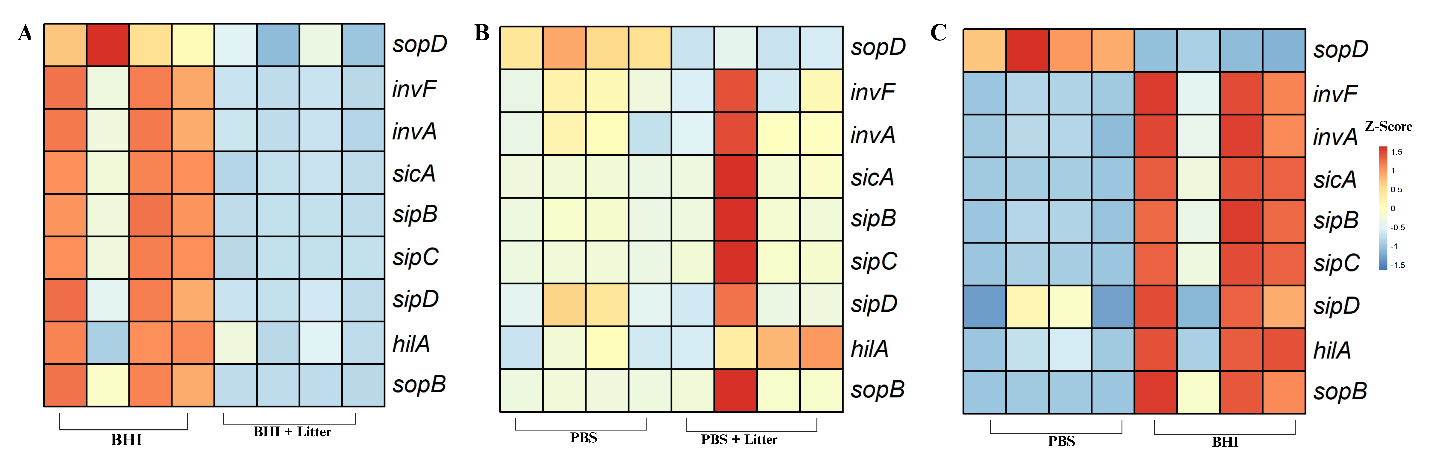


**Supplementary Figure 10**. **Heatmaps of differentially expressed genes in Salmonella Infantis across treatment conditions for virulent genes.**

RNA-seq was performed with four biological replicates per treatment, DESeq2 log₂ fold-change values were used to get upregulated and downregulated genes. For the creation of heatmap, genes were grouped by functional categories, and heatmaps were generated using pheatmap R package with row-wise z-score scaling, so that each gene’s expression is normalized relative to its mean and standard deviation across samples. Color intensity reflects relative expression: read indicates expression above gene’s average, blue indicates expression below the gene’s average. (A) BHI vs. BHI + Litter, (B) PBS vs. PBS + Litter, and (C) PBS vs. BHI represent three pairwise comparisons made. Columns represent experimental conditions, and each row virulence genes.
